# Measuring the Quality of University Lectures: Development and Validation of the Instructional Skills Questionnaire (ISQ)

**DOI:** 10.1371/journal.pone.0149163

**Published:** 2016-02-26

**Authors:** Mariska H. Knol, Conor V. Dolan, Gideon J. Mellenbergh, Han L. J. van der Maas

**Affiliations:** Department of Psychological Methods, University of Amsterdam, Amsterdam, The Netherlands; University of Geneva, SWITZERLAND

## Abstract

In higher education, student ratings are often used to evaluate and improve the quality of courses and professors’ instructional skills. Unfortunately, student-rating questionnaires rarely generate specific feedback for professors to improve their instructional skills. The impact of student ratings on professors’ instructional skills has proven to be low. This study concerns the psychometric properties of the Instructional Skills Questionnaire (ISQ), a new theory-based student-rating-of-teaching questionnaire with specific questions concerning lecturing skills. The ISQ is administered after a single lecture. This way, it serves as a formative feedback instrument for university professors *during* courses to assist them to improve and (re-) evaluate their skills if necessary. The ISQ contains seven dimensions of professors’ instructional skills and three student (self perceived) learning outcomes. In this study, Dutch students in 75 courses rated three 90-minute lectures (T1, T2 and T3) of their respective professors using the ISQ. In total, 14,298 ISQ-forms were used to rate 225 lectures. The teacher level reliabilities of the seven dimensions were found to be good at each measurement occasion. In addition, confirmatory multilevel factor analysis confirmed a seven dimensional factor structure at the teacher level at each measurement occasion. Furthermore, specific teacher level factors significantly predicted students’ (self-assessed) learning outcomes. These results partly supported the proposed theoretical framework on the relationship between the ISQ teaching dimensions and the student learning process, and provided evidence for the construct validity of the instrument. In sum, the ISQ is found to be a reliable and valid instrument, which can be used by professors and faculty development centers to assess and improve university teaching.

## Introduction

Students in higher education are requested to rate the quality of courses and professors on a regular basis. Most often, student-rating questionnaires are designed to evaluate complete courses, and are administered at the end of the course or term. To collect student ratings on each course, year after year, has become an extensive practice. One important aim of collecting student ratings is to provide feedback to professors to improve the quality of their teaching. Unfortunately, the impact of student ratings collected at the end of the term or course on professors’ instructional skills is small to null [1, 2]. Marsh [2] investigated a cohort of 195 teachers, who were evaluated continuously over 13 years, and found no overall increase of student ratings of teaching over time.

This lack of impact is related to the nature of the feedback. According to the literature, for feedback to be effective, it should be well-timed, specific, reliable, and should facilitate realistic behavioural change [3, 4]. Often course evaluations do not meet these requirements. First, student feedback collected at the end of a term or course is arguably ill-timed feedback. Most of the time, professors are not able to use the feedback until the next semester or year. Studies have showed that intermediate (often mid-term) feedback is more effective than end-of the term feedback, in terms of an increase in student ratings of teaching over time [5]. Combining such intermediate feedback with (expert) consultation has proven to be even more effective [6, 7]. However, standard course evaluation questionnaires are not automatically suited for intermediate use. For example, questions related to examination, or expected grades do not apply. Also, standard course evaluation questionnaires are often not designed to provide extensive feedback. This brings us to the second issue with course evaluations as a source of feedback for professors. As common student-rating questionnaires are designed to evaluate complete courses, the number of specific questions on the professors’ instructional skills is often limited. In some cases, course evaluations are limited to a single question (e.g., ‘how do you rate your professor?’). A limited number of questions often does not reflect or reliably measure the multidimensionality of instructional skills (see below on the dimensions of teaching).

The present study concerns the development and psychometric analyses of a new research-based student-rating-of teaching questionnaire, the Instructional Skills Questionnaire (ISQ). The ISQ is administered immediately after a single lecture *during* a course and contains detailed questions on seven dimensions of instructional skills. The present study we investigated the reliability, factor structure, and the construct validity of the ISQ.

This instrument can be used to provide professors with specific, well-timed, and relevant feedback on various aspects of their teaching. It enables them to assess, improve, and re-assess their instructional skills during their course. The ISQ is also useful for faculty development centers and consultants, as a source of intermediate feedback. Such feedback with additional consultation has proven to be highly effective.

Below, we first present the theoretical background of the ISQ dimensions of effective teaching behaviour. A research-based approach to the development of the questionnaire enhances the content validity of the ISQ, and informs our expectations concerning the factor structure of the ISQ. Furthermore, we discuss the expected relationships between the ISQ teaching dimensions and the student learning process. This provides a conceptual framework concerning the construct validity of the ISQ. Next, we present our findings concerning the reliability, internal structure and construct validity of the ISQ. We conclude this paper with a discussion of the psychometric properties of the ISQ.

### Dimensions of Teaching

There is an extensive body of research concerning student ratings of courses and professors in higher education (for an overview see [8–10]). These student ratings have proven to be reliable and stable, reasonably valid (as judged by a variety of indicators of effective teaching), and relatively unbiased [8].

Student ratings of courses and professors are known to be multidimensional. Feldman differentiated twenty to twenty-eight teaching dimensions, based on students’ views on effective teaching, on student ratings, and on content analyses of single items and multiple-item scales found in the higher education research literature ([11–16]. Feldman [16] related these dimensions to domains of student achievement and overall evaluations. He found the dimensions most highly related to both domains to be 1) teacher clarity and comprehensibility; 2) teacher stimulation of interest in the subject matter; 3) perceived outcome or impact of instruction; and 4) teachers’ preparation (organization of the course). In addition, many student-rating questionnaires have been developed using factor analytic (empirical) or theory-based approaches. The development and study of two instruments (the SEEQ and the Evalec) formed the theoretical basis of a Dutch instrument (the UvAlon), which in turn formed the basis of the ISQ. We discuss the ISQ background in this order.

First, a thoroughly investigated instrument is the Students' Evaluation of Education Quality (SEEQ), developed by Marsh and colleagues [17–19]. The SEEQ includes nine dimensions of course and teaching effectiveness: 1) Organization/Clarity (clear course design, preparations, and instructions), 2) Breath of Coverage (elaboration on the subject matter), 3) Instructor Enthusiasm (maintaining the interest of students during class), 4) Individual Rapport (accessibility and genuine interest in students), 5) Group Interaction (encouragement of class participation and discussion), 6) Learning/Value (learn valuable content, increase interest, etc.) 7) Examinations/Grading (fair grading and appropriate examination), 8) Assignments/Readings (effective assignments), and 9) Difficulty/Workload. Some dimensions are related to teaching behaviour (dimension 2, 3, 4, 5), some are related to course design (dimensions 7, 8, 9), and some are related to both teaching behaviour and course design (dimensions 1 and 6). The reliability and validity of the SEEQ has been established in different settings [20–22].

Second, De Neve and Janssen [23] developed the Evalec (EVAluation of LECturing). The authors took a theoretical approach by focusing on specific lecturing behaviours, which facilitate the student learning process. The five Evalec dimensions (Validating, Stimulating, Conversation, Directing, and Structuring) were designed to address different lecture components, according to Van Gelder’s model for didactic analysis [24]. These components include introducing clear objectives, tuning in to the students entry level and interests, applying effective teaching-learning strategies (e.g., clear exposition, well-selected content, useful learning aids, eliciting discussions), and evaluating the outcome.

Third, based on the work of Feldman, the SEEQ, and the Evalec, Vorst and Van Engelenburg [25] developed a Dutch course evaluation instrument for the University of Amsterdam, the Uvalon. The Uvalon included six dimensions on *course characteristics*, seven dimensions of *teaching behaviour*, and two dimensions on *student behaviour*. The psychometric properties of the Uvalon were investigated in several internal reports of the University of Amsterdam [25–27].

Given the Uvalon’s theoretical and empirical foundation, the ISQ was based on Uvalon’s seven dimensions of *teaching behaviour* (Structure, Explication, Stimulation, Validation, Instruction, Conversation and Interaction). Since the ISQ is meant to evaluate single lectures and to serve as a formative feedback instrument for professors, we retained only the dimensions pertaining to teaching behaviour. We adjusted and replaced some of the outdated items to obtain a more up-to-date representation of university lectures. Finally, we renamed the dimensions Conversation and Interaction to Comprehension and Activation, respectively, to more accurately convey their meaning. We hypothesized that both dimensions reflect interaction between the professor and the students, but with different purposes. The items of Conversation dimension focus on providing occasion for students to ask questions, and for the professor to check whether students understand the subject matter (hence the new label Comprehension). Thus, the purpose is the consolidation of comprehension of the subject matter during the lecture. The items of the Interaction dimension focus on fostering the students’ active involvement (hence the label Activation). In sum, the seven ISQ dimensions defined as follow:

1. Structure: the extent to which the subject matter is handled systematically and in an orderly way
2. Explication: the extent to which the instructor explains the subject matter, especially the more complex topics
3. Stimulation: the extent to which the instructor interests students in the subject matter
4. Validation: the extent to which the instructor stresses the benefits and the relevance of the subject matter for educational goals or future occupation
5. Instruction: the extent to which the instructor provides instructions about how to study the subject matter
6. Comprehension: the extent to which the instructor creates opportunities for questions and remarks regarding the subject matter
7. Activation: the extent to which the instructor encourages students to actively think about the subject matter

To indicate the relationship between the instruments, the dimensions of the SEEQ, Evalec, Uvalon and the ISQ are listed in Table 1. In addition, the relationship with Feldman’s categories is indicated. In terms of content validity, the relationship with Feldman’s categories and the other instruments show that the Uvalon / ISQ teaching dimensions contain the most important teaching behaviours, according to previous research.

**Table 1:** Dimensions of the evaluation instruments SEEQ, Evalec, Uvalon, ISQ and the relationship with Feldman's categories.

SEEQ	Evalec	Uvalon	ISQ	Feldman's Categories
(Marsh, 1984, 1987)	(De Neve & Janssen, 1982)	(Vorst & Van Engelenburg, 1992)		(1976, 2007)
		*Teaching behaviour*		
Organization / Clarity	Structuring	Structure	Structure	Teacher’s Preparation; Organization of the Course (I)
Breath of Coverage		Explication	Explication	Clarity and Understandableness (I)
				Teacher’s Knowledge of Subject Matter (I)
				Teacher’s Intellectual Expansiveness (I)
Instructor Enthusiasm	Stimulating	Stimulation	Stimulation	Teacher’s Stimulation of Interest in the Course and Its Subject Matter (I)
				Teacher’s Enthusiasm (for Subject or for Teaching) (I)
(Learning / Value)	Validating	Validation	Validation	Nature and Value of the Course Material (Including Its Usefulness and Relevance) (III)
(Organization / Clarity)	Directing	Instruction	Instruction	Clarity of Course Objectives and Requirements (III)
Individual Rapport	Conversation	Conversation	Comprehension	Teacher’s Sensitivity to, and Concern with, Class Level and Progress (II)
				Teacher’s Availability and Helpfulness (II)
				Teacher’s Concern and Respect for Students (II)
Group Interaction		Interaction	Activation	Intellectual Challenge and Encouragement of Independent Thought (by the Teacher and the Course) (II)
				Teacher’s Encouragement of Questions and Discussion, and Openness to Opinions of Others (II)
		*Course characteristics*		
Learning / Value		Learning/Value		Perceived Outcome or Impact of Instruction (III)
Examinations / Grading		Examination		Teacher’s Fairness; Impartiality of Evaluation of Students; Quality of Examinations (III)
				Nature Quality, and Frequency of Feedback from the Teacher to Students (III)
Assignments / Readings		Literature		Nature and Usefulness of Supplementary Materials and Teaching Aids (III)
Difficulty / Workload		Workload /Difficulty		Difficulty of the Course (and Workload) (III)
				Difficulty of the Content (and Workload) (III)

*Notes*: Feldman (1976b) clustered the categories in Presentation (I), Facilitation (II), and Regulation (III). This is indicated in parentheses following each category. Categories not included are: Teacher’s Elocutionary Skills, Personality Characteristics (“Personality”) of the Teacher, Teacher Motivates Students to Do Their Best, Teacher’s Encouragement of Self-Initiated Learning, Teacher’s Productivity in Research Related Activities, Classroom Management, Pleasantness of Classroom Atmosphere, Individualization of Teaching, Teacher Pursued and/or Met Course Objectives.

Like De Neve and Janssen in the development of the Evalec, we adopt a theoretical perspective on the relationship between the selected teaching dimensions, and the manner in which they facilitate the student's learning process. In the next section, we present the theoretical framework for the ISQ.

### Relating teaching behaviour to student learning

Based on the literature, Vermunt [28] distinguished three domains of activities relevant to students’ learning: cognitive, affective, and regulative learning activities. Cognitive activities serve to process learning content (e.g., looking for relations among parts of the subject matter, thinking of examples). These activities lead directly to learning. Affective learning activities are directed at coping with the feelings that arise during learning, and lead to an emotional state that may positively, neutrally, or negatively affect the learning process (e.g., motivating oneself). Regulative learning activities are directed at regulating the cognitive and affective learning activities, and therefore indirectly facilitate learning results (e.g., orienting on a learning task). Vermunt and Verschaffel [29] noted great similarities between these learning activities and teaching activities, as found in the literature. They adopted the terms learning functions and teaching functions. *Learning functions* are categorized into cognitive/processing, affective, and regulation functions (parallel to the distinction between learning activities). *Teaching functions* refer to teaching behaviour that promotes student learning. Some examples are a) presenting and clarifying the subject matter promotes cognitive learning functions, b) creating and maintaining a positive motivational and emotional climate for students promotes affective learning functions, and c) guiding students’ learning processes promotes regulation learning functions [29].

**Fig 1:**
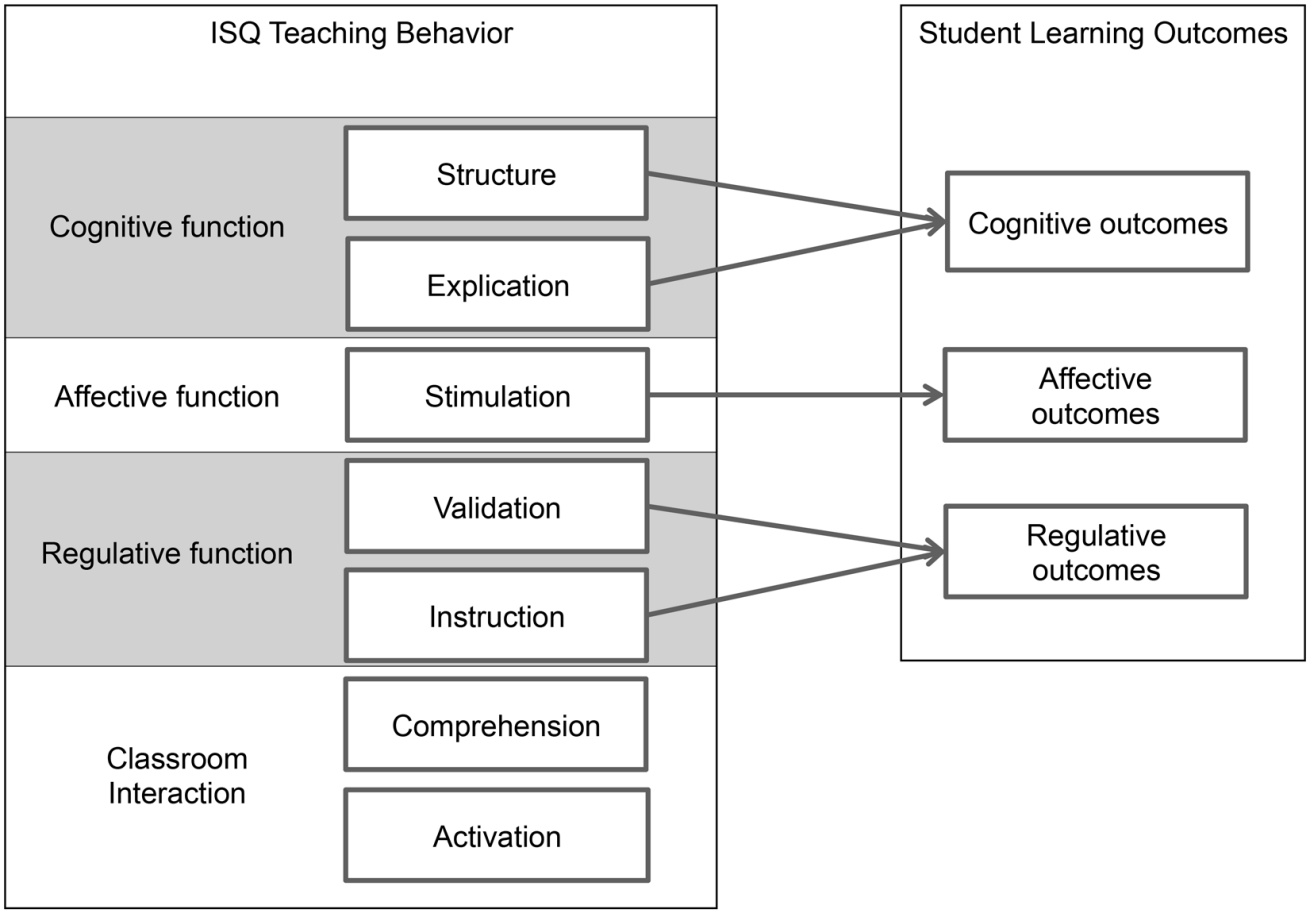
Conceptual framework on the relationship between the seven ISQ dimensions on teaching behaviour and student learning outcomes.

We propose that the teaching functions are closely related to the ISQ teaching dimensions. A representation of the hypothesized relationships between teaching behaviour and student learning outcomes is given in Fig 1.

In the present study, we investigated this conceptual framework by analyzing the relationships between the ISQ dimensions and student perceptions of their cognitive, affective, and regulative learning outcomes. We interpreted the results in terms of our conceptual framework and the construct validity of the ISQ.

### Research Questions

In sum, the present study aims to address the following research questions:

1. What are the psychometric properties of the ISQ?
  - Reliability: are the seven ISQ dimensions of teaching behaviour reliable?
  - Factor structure: does the theory-based ISQ seven-factor model provide an adequate account of the covariance structure?
  - Construct validity: what is the relationship between students’ perceptions of their cognitive, affective, and regulative learning outcomes variables and the seven ISQ dimensions of teaching behaviour?

We used a confirmatory approach to investigate question a and b, as the seven ISQ dimensions were research-based, theory-driven and piloted (see methods section). We used an exploratory approach to investigate question c, as we started with a conceptual framework to answer this research question.

## Method

### Participants

#### Professors

In total, 95 university professors from five departments of a Dutch university were scheduled to give a minimum of 3 lectures during 95 courses in 2009–2010. Of the 95 professors, 87 professors agreed to participate. Of the 87, 12 professors dropped out due to circumstance beyond their control (e.g., illness, rescheduling). This resulted in a final sample of 75 professors (63 male, 12 female, age *M* = 46.8, *SD* = 9.6) from the departments of Law (*N* = 20), Economics (*N* = 24), Science (*N* = 13), Social and Behavioural Sciences (*N* = 13), and Humanities (*N* = 5). Of the 225 lectures (3 lectures per professor) that were scheduled to be rated by the students, 7 lectures were inadvertently not rated. This resulted in 73 rated lectures at the first measurement occasion (T_1_), 74 rated lectures at the second measurement occasion (T_2_), and 71 rated lectures at the third measurement occasion (T_3_).

#### Students

The students in the selected courses rated their professors by completing the Instructional Skills Questionnaire (ISQ) immediately after three lectures during the course. In total, the ISQ was completed 14,596 times: 6,009 times at the first measurement occasion (T_1_), 4,766 times at the second measurement occasion (T_2_) and 3,821 times at the third measurement occasion (T_3_). There was an expected decrease in attendance over time, as a number of students invariably drop out during courses.

A mean response rate of 90.2% was observed in 76 randomly selected lectures. The mean class size, in terms of ISQ forms completed, was 82.3 students at T1 (*SD* = 64.9, *min* = 13, *max* = 365), 64.4 students at T2 (*SD* = 49.8, *min* = 13, *max* = 222), and 53.8 students on T3 (*SD* = 43.1, *min* = 8, *max* = 206). The class sizes per professor per measurement occasion are listed in S2 Table.

### Measures

The Instructional Skills Questionnaire (ISQ) includes seven dimensions of lecturing skills, as measured by 28 items (4 items x 7 dimensions; the items are listed in Table 2). The Dutch version of the ISQ and the order in which the items were administered is presented in S1 Table. Each dimension was measured by two indicative items and two contra-indicative items with a 7-point Likert scale response format (response options ranging from strongly disagree to strongly agree). The contra-indicative items were recoded prior to analyses.

**Table 2:** English translation of the Dutch Instructional Skills Questionnaire (ISQ).

**Structure:**	**The extent to which the subject matter is handled systematically and in an orderly way**
Str 1	The lecture has a clear structure
Str 2	The instructor gives clear summaries
Str 3	The subject matter is presented incoherently
Str 4	The lecture is unorganized
**Explication:**	**The extent to which the instructor explains the subject matter, especially the more complex topics**
Expl 1	The instructor explains the subject matter clearly
Expl 2	The instructor is unclear
Expl 3	The instructor’s explanations are hard to follow
Expl 4	The instructor gives clarifying examples
**Stimulation:**	**The extent to which the instructor** interests students in the subject matter
Stim 1	The lecture is boring
Stim 2	The instructor enlivens the subject matter
Stim 3	It is hard to stay focused on the lecture
Stim 4	The instructor interests you in the subject matter
**Validation:**	**The extent to which the instructor stresses the benefits and the relevance of the subject matter for educational goals or future occupation**
Val 1	Little is said about the application of the subject matter
Val 2	The instructor indicates the relevance of the subject matter
Val 3	The utility of the subject matter is hardly discussed
Val 4	The instructor shows why the subject matter is important
**Instruction:**	**The extent to which the instructor provides instructions about how to study the subject matter**
Instr 1	The instructor is unclear about which aspects of the subject matter are important
Instr 2	It is often unclear what the main and side issues are
Instr 3	It is clear what the instructor requires of me
Instr 4	The instructor indicates which parts of the subject matter are essential
**Comprehension:**	**The extent to which the instructor creates opportunities for questions and remarks regarding the subject matter**
Comp 1	The instructor provides insufficient occasion to ask questions
Comp 2	The instructor encourages students to ask questions about the subject matter
Comp 3	The instructor checks whether students understand the subject matter
Comp 4	The instructor hardly addresses the students’ comments
**Activation:**	**The extent to which the instructor encourages students to actively think about the subject matter**
Act 1	Students are encouraged to think along during the lecture
Act 2	The instructor provides little opportunity for discussions
Act 3	During this lecture there is hardly any occasion to discuss the subject matter
Act 4	The instructor involves students in the lecture
**Student Learning Outcomes**	
Cognition:	I learned a lot from this lecture
Affection:	Because of this lecture, I want to learn more about the subject matter
Regulation:	Because of this lecture, I now know what I have yet to study

The items were partly adapted from Vorst and van Engelenburg’s [25] Uvalon items. From the pool of Uvalon items, 28 items concerning lecturing behaviour were selected, rewritten and (or) added to reflect the seven dimensions. Prior to this study, the ISQ items were piloted at the psychology department of a Dutch university. The questionnaire was administered at the end of twenty-five lectures of twenty-five different professors. In total, 609 forms were completed. Cronbach’s alphas for the seven dimensions on teachers’ mean ratings were .66 for Structure, .76 for Explication, .93 for Stimulation, .84 for Validation, .72 for Instruction, .88 for Comprehension and .95 for Activation. In this pilot version, a 5-point likert scale was used. With SET ratings, the variation is often small (see for example similar SET variance in [30, 31]). To increase resolution, we used a 7-point likert scale in the final version of the instrument. A few items were adapted to improve the reliability of the subscales and items. Finally, three items were added to the questionnaire to measure the students’ perception of their cognitive, affective, and regulative learning outcomes (listed in Table 2).

In total, 3.7% of the item responses were missing. We worked with the raw data, thus we did not impute missing data, list-wise delete forms with missing data, delete outliers, or apply any other data cleaning techniques.

### Procedure

Prior to the start of their courses, all professors received procedural instructions by email. Professors informed their students by a standardized email that they (i.e., the professors) would be participating in a research project on the quality of the lectures at the university. Students were invited to participate by rating three lectures during the course. A standard course at this university took eight weeks, with one or two lectures per week. Students rated the lectures in week 2 (T1), week 4 (T2) and week 6 (T3).

A standard lecture at this university took 90 minutes (with a 15-minute break after 45 minutes). In the final fifteen minutes of the lecture, professors reserved five minutes for students to rate the lecture. At that moment, research assistants entered the classroom and distributed the ISQ-forms. Students were instructed to focus on the current lecture, while completing the ISQ-form. They were asked to provide their student ID number for research purposes, and were assured of confidentiality by means of an extra statement on the ISQ-form (statement: ISQ-reports contain anonymous results on class level, no individual results are reported back to professors). Research assistants collected the ISQ-forms and processed the data. Professors were not able to link feedback to any individual student at any point in this study.

The professors were randomly assigned to one of three conditions; a feedback-only condition, in which professors received the student feedback each time shortly after the rated lecture so they could improve their upcoming lectures (N = 24); a feedback-plus-consultation condition, in which professors received student feedback and collaborative consultation with a consultant after each rated lecture to improve the subsequent lecture (N = 26); and a control condition, in which professors received the student feedback at the end of the course (N = 25). The interventions took place after the first measurement occasion. The impact of these experimental conditions on student ratings over time was significant. Professors in the feedback-plus-consultation condition showed a significant improvement in student ratings over time compared to professors in the other two conditions. These analyses and results are discussed in detail in a separate paper (under submission). In the present study, we focus on the psychometric characteristics of the ISQ.

#### Ethics statement

The current study did not concern manipulation or involve deception, and participation was completely voluntary. It was not in any way harmful to participants, and mainly provided university professors with extra means to improve their teaching. Participants were free to withdraw at any time. Written statements were sent to all potential participants at the outset of the study. Directors and professors were approached in person by the first author. All directors gave oral permission to the first author to approach their department’s professors. The directors announced the study to faculty by email and emphasized the fact that participation was voluntary. The first author then approached all professors and explained the study in person. The first author furthermore explained that participation was voluntary, but random assignment to the conditions was mandatory. Professors gave their oral consent to the first author and provided their background information, course information and course schedules. These forms are archived. Professors informed their students with a standard announcement provided by the first author, stating student participation was voluntary. In addition, the ISQ-form included the statement that the data would be used for research purposes. To increase confidentiality, teacher- and student numbers were assigned to the final dataset. Students were assured of confidentiality by a statement included in the ISQ form. Professors were assured of confidentiality in person by the first author when they were recruited and in writing on all documentation and evaluation forms (which contained their assigned teacher number).

### Statistical Analyses

In this study, students rated three lectures per professor, giving rise to dataset at each measurement occasion (denoted T1, T2, and T3). The first measurement occasion was used to investigate the three research questions. The second and third measurement occasion were used to quasi-cross-validate the results.

#### Nested data

Each research question was investigated by means of multilevel analyses. With this approach we took the nested structure of the data into account [32, 33]. Student ratings datasets are characterized by a dependent (nested) structure. Specifically, students’ ratings (level 1: student level) are nested in professors (level 2: teacher level). Ignoring this nested structure may result in biased estimates and tests [32, 33]. To our knowledge, multilevel analyses have not yet been applied to student evaluations of a single lecture (for an example on course evaluations, see [34]). We used Mplus software [35] to perform all multilevel analyses. Below, we elaborate on each analysis per research question.

#### Descriptive information

The descriptives were computed per ISQ item at the teacher level (level 2) and the student level (level 1) at each measurement occasion (T1, T2, and T3). Using multilevel confirmative factor analysis (MCFA), we estimated the mean on each item, the variance at the teacher level and the student level, at each measurement occasion. The estimated variance at the teacher level represented the differences between professors in their mean student ratings. The estimated variance at the student level represented the differences between students’ ratings of their professors. As a baseline model, we specified a fully saturated covariance structure model in which we merely estimated the teacher level and student level covariance matrices. We computed the intra-class correlations (ICCs) for each item at each measurement occasion, to obtain a measure of item-level clustering. The ICCs represent the variance of the ratings between professors (level 2) divided by the total variance of the ratings for each item, and provide an indication of the strength of the clustering.

#### Reliability of the ISQ

Geldhof, Preacher and Zyphur [36] discuss the calculation of reliability in a two level design. It is important to distinguish between student level and teacher level reliability as these need not be equal. We computed Cronbach's alphas on level 1 and 2 by applying Cronbach’s equation separately to the student level (level 1) and teacher level (level 2) covariance matrices, for each subscale comprising 4 items (for details, see [36]). We applied the same procedure to the results obtained with the datasets of T2 and T3 to obtain the reliabilities at these occasions. To obtain the student level and teacher level covariance matrices, we fitted a saturated two level model to each set (of 4) items at occasions T1, T2, and T3. This model was saturated in the sense that the student and teacher level covariance matrices were estimated freely. As the focus is on the teacher level results, we report the teacher level reliabilities here. We include the student level reliabilities in S3 Table (at the request of a reviewer).

#### Factor structure of the ISQ

Often, the internal structure of a questionnaire may be studied by means of exploratory or confirmatory factor analysis. Given the strong theoretical and empirical basis of the ISQ dimensions, we used confirmatory factor analyses of the ISQ. We investigated the psychometric properties of the ISQ, in terms of its ability to differentiate between professors on seven theory-driven dimensions.

In terms of *multilevel* factor analyses on the ISQ, the level 1 (student level) factor model reflected differences between students in their ratings of their professor. The level 2 factor model (teacher level) reflected the differences between the professors in the average responses of their students. Since student-rating instruments were meant to differentiate between professors on the given dimensions, the teacher-level results were of primary interest in investigating the factor structure [8, 18, 37–39]. However, as teacher level data were based on student ratings, the ISQ necessarily provided student level data. With multilevel factor analyses we studied the structure of the ISQ at the teacher level (level 2), while taking the variance at the student level (level 1) into account. To this end, we performed a MCFA on the data of T1 with the expected seven-factor model at the teacher level (level 2), and an unconstrained model at the student level (level 1). By an unconstrained model we mean that we estimated the covariance structure of the data at the student level without constraints (i.e., we adopted a saturated level 1 covariance structure model)

To take into account the experimental conditions (see procedure), two dummy coded variables were added to the model as teacher-level covariates; C1 (feedback-only condition: coded 1, control and feedback-plus-consultation condition: coded 0) and C2 (feedback-plus-consultation condition: coded 1, control and feedback-only condition: coded 0). At measurement occasion one, these dummy covariates were irrelevant, because the interventions took place after the first measurement occasion. However, at occasions T2 and T3, it is important to accommodate possible differences between the conditions.

Finally, to cross-validate the hypothesized seven-factor model, we fitted the same model with MCFA on T2 data and T3 data (again, a more accurate term is “quasi-cross-validation”, given the repeated measures). According to Schermelleh-Engel, Moosbrugger, and Muller [40] a good fit is represented by the following fit indices: Root Mean Square Error of Approximation (RMSEA) < 0.05, Standardized Root Mean Square Residual (SRMR) < 0.05, Comparative Fit Index (CFI) > 0.97, Tucker-Lewis-Index (TLI) > 0.97. Acceptable fit indices are: RMSEA < 0.08, SRMR < 0.10, CFI > 0.95, TLI > 0.95. In addition, item loadings should be significant (p < .01). We used these fit indices to verify the hypothesized teacher level seven-factor model at each measurement occasion.

In using MCFA, we were aware of the relatively small sample size at the teacher level. We do note that in this case teacher level data did not concern isolated data points, but data based on multiple student observations, which increased the resolution and reliability. This results in a highly reliably estimate of the covariance matrix.

#### Construct validity of the ISQ

Multilevel regression analyses allowed us to investigate the relationship between teacher-level factors (the seven specific ISQ dimensions of teaching behaviour) and students’ self-assessed learning outcomes in the data collected at T1. In this multilevel regression model, we assigned the seven ISQ dimensions as independent latent (teacher level) variables and the learning outcome variables Cognition, Affection, and Regulation as dependent variables. The regression analysis was carried out at the teacher level, i.e., we regressed the means of the learning outcome variables (the means of the students nested under the same professor) on the teacher level factors. As in the previous analyses, we left the covariance structure of the ISQ data unconstrained at the student level, to accommodate the presence of the student level covariance structure. As in the previous analyses, the two dummy coded variables (C1 and C2) were added to the model as teacher-level covariates, to take the experimental conditions into account (see procedure).

We used an exploratory approach by regressing each dependent variable on each independent variable. We applied the Holm-Bonferroni step-down procedure to correct for multiple testing. Finally, to quasi-cross-validate the results, the same analyses were performed on T2 data. We did not fit this model to the T3 data in view of the relatively small sample size (owing to attrition).

## Results

### Descriptive Information

A descriptive overview per ISQ item at the teacher level (mean student ratings per professor and variance between these means) and the student level (variance between students) at each measurement occasion (T1, T2 and T3) is presented in Table 3. Visual inspection of the data revealed slight skewness of the item scores at each measurement occasion, at both the student and the teacher level. As the skewness never exceeded an absolute value of 1, we treated the data as normally distributed.

**Table 3:** ISQ descriptive information—measurement occasion one, two and three.

		T1				T2				T3			
ISQ		Teacher level		Student level		Teacher level		Student level		Teacher level		Student level	
Scale	Item	Mean	Variance	Variance	ICC	Mean	Variance	Variance	ICC	Mean	Variance	Variance	ICC
**Structure**	**Str1**	5,27	0,17	1,25	0,12	5,29	0,18	1,28	0,12	5,29	0,20	1,28	0,14
	**Str2**	4,74	0,13	1,47	0,08	4,83	0,15	1,53	0,09	4,86	0,16	1,52	0,09
	**Str3**	5,33	0,18	1,45	0,11	5,29	0,19	1,46	0,12	5,25	0,20	1,55	0,11
	**Str4**	5,39	0,22	1,52	0,13	5,38	0,22	1,44	0,13	5,29	0,25	1,51	0,14
**Explication**	**Expl1**	5,29	0,19	1,18	0,14	5,25	0,23	1,20	0,16	5,23	0,19	1,24	0,13
	**Expl2**	5,44	0,21	1,60	0,12	5,35	0,22	1,67	0,12	5,31	0,23	1,68	0,12
	**Expl3**	5,33	0,33	1,49	0,18	5,25	0,32	1,54	0,17	5,16	0,28	1,68	0,14
	**Expl4**	5,43	0,24	1,34	0,15	5,44	0,26	1,34	0,16	5,39	0,25	1,41	0,15
**Stimulation**	**Stim1**	4,59	0,57	1,92	0,23	4,67	0,65	1,89	0,26	4,73	0,62	1,88	0,25
	**Stim2**	4,87	0,63	1,53	0,29	4,94	0,74	1,54	0,32	4,95	0,62	1,53	0,29
	**Stim3**	4,42	0,64	2,06	0,24	4,43	0,70	2,03	0,26	4,43	0,60	2,10	0,22
	**Stim4**	4,70	0,47	1,51	0,24	4,76	0,56	1,51	0,27	4,76	0,56	1,56	0,26
**Validation**	**Val1**	4,67	0,16	1,86	0,08	4,79	0,18	1,85	0,09	4,81	0,12	0,80	0,06
	**Val2**	4,87	0,11	1,41	0,07	4,93	0,16	1,38	0,10	4,94	0,13	1,40	0,09
	**Val3**	5,06	0,17	1,66	0,09	5,13	0,20	1,49	0,12	5,09	0,18	1,52	0,10
	**Val4**	4,70	0,16	1,37	0,10	4,80	0,18	1,45	0,11	4,81	0,15	1,43	0,10
**Instruction**	**Instr1**	4,83	0,10	2,20	0,05	4,75	0,11	2,36	0,04	4,68	0,09	2,46	0,04
	**Instr2**	4,72	0,09	1,80	0,05	4,72	0,11	1,85	0,06	4,69	0,10	1,99	0,05
	**Instr3**	4,49	0,11	1,64	0,06	4,57	0,13	1,62	0,08	4,61	0,11	1,71	0,06
	**Instr4**	4,77	0,15	1,51	0,09	4,86	0,22	1,47	0,13	4,86	0,18	1,58	0,10
	**Comp1**	5,39	0,27	1,92	0,13	5,30	0,22	2,01	0,10	5,21	0,20	2,12	0,08
	**Comp2**	4,41	0,54	1,53	0,26	4,49	0,50	1,51	0,25	4,58	0,55	1,59	0,26
	**Comp3**	4,67	0,31	1,59	0,16	4,72	0,32	1,59	0,17	4,74	0,36	1,65	0,18
	**Comp4**	5,60	0,24	1,33	0,15	5,53	0,21	1,34	0,14	5,49	0,19	1,48	0,12
**Activation**	**Act1**	4,75	0,57	1,51	0,27	4,80	0,54	1,54	0,26	4,82	0,47	1,54	0,24
	**Act2**	4,85	0,35	1,58	0,18	4,91	0,32	1,64	0,16	4,96	0,27	1,61	0,15
	**Act3**	4,83	0,47	1,83	0,20	4,93	0,39	1,81	0,18	1,98	0,42	1,78	0,19
	**Act4**	4,74	0,66	1,49	0,31	4,80	0,59	1,47	0,29	4,84	0,48	1,49	0,24

Notes: T1 = measurement occasion one. T2 = measurement occasion two. T3 = measurement occasion three. ICC = intra-class correlation

The intra-class correlations of the items varied between 0.04 and 0.32, with a mean of 0.15 (T1, T2 and T3 combined). This indicated that on average 15% of the variance of the ratings was due to mean differences between the professors.

### Reliability of the ISQ

Teacher level alphas of each subscale at each measurement occasion are listed in Table 4. Student level alphas of each subscale at each measurement occasion are listed in S3 Table. The reliability of the subscales at the teacher level was high, with a mean alpha of .94 at T1, a mean alpha of .97 at T2, and a mean alpha of .96 at T3. The reliabilities at the teacher level are of main interest as they pertain to the dimensions.

**Table 4:** ISQ reliability at the teacher level—measurement occasion one, two and three.

	T1	T2	T3
ISQ scale	α	α	α
**Structure**	0,93	0,96	0,96
**Explication**	0,95	0,97	0,96
**Stimulation**	0,98	0,99	0,99
**Validation**	0,94	0,96	0,96
**Instruction**	0,90	0,97	0,95
**Comprehension**	0,93	0,94	0,95
**Activation**	0,97	0,97	0,98

Notes: T1 = measurement occasion one, T2 = measurement occasion two, T3 = measurement occasion three. α = Cronbach’s alpha

### Factor structure of the ISQ

The first multilevel confirmatory factor analysis (MCFA at T1) included the expected seven-factor model at the teacher level, and an unconstrained (covariance structure) model at the student level. The model yielded a good fit, as indicated by the goodness-of-fit indices (T1: RMSEA = 0.021, SRMR between = 0.090, CFI = 0.977, TLI = 0.950). Three residual variances were fixed to zero, as they assumed small negative values, resulting in loadings of 1 (items str3, expl1 and stim1). All the factor loadings were statistically significant at the 1% significance level, with a mean standardized loading of .92 (min = 0.57, max = 1.00, median = 0.96).

The good fit of this model was confirmed at the second measurement occasion (T2: RMSEA = 0.022, SRMR between = 0.080, CFI = 0.978, TLI = 0.952) and the third measurement occasion (T3: RMSEA = 0.025, SRMR between = 0.072, CFI = 0.976, TLI = 0.958). Again, all the factor loadings were statistically significant, with a mean loading of .95 at both the second and third measurement occasion (T2: *min* = 0.78, *max* = 1.00, *median* = 0.97, T3: *min* = 0.79, *max* = 1.00, *median* = 0.97). Again, three residual variances were fixed to zero, resulting in loadings of 1 (at occasion T2: items str3, stim1 and instr1, at occasion T3: items str3, val4, and act2). Table 5 presents the goodness-of-fit indices at each measurement occasion. Table 6 presents factor loadings on teacher level at each measurement occasion are presented.

**Table 5:** MCFA goodness-of-fit indices—measurement occasion one, two and three.

Measurement occasion	Student level	Teacher level	df	RMSEA	CFI	TLI	SRMR student level	SRMR teacher level
T1	unconstrained	7 factors	374	0,021	0,977	0,950	0,001	0,090
T2	unconstrained	7 factors	374	0,022	0,978	0,952	0,001	0,080
T3	unconstrained	7 factors	374	0,025	0,976	0,958	0,001	0,072

Notes: At each measurement occasion, the model contained the following seven factors at the teacher level: Structure, Explication, Stimulation, Validation, Comprehension and Activation. The model was unconstrained at the student level. Correction was made for Condition. Df = degrees of freedom. RMSEA = Root Mean Square Error of Approximation. CFI = Comparative Fit Index. TLI = Tucker-Lewis-Index. SRMR = Standardized Root Mean Square Residual.

**Table 6:** ISQ item factor loadings—measurement occasion one, two and three.

	T1	T2	T3
	Estimate(S.E.)	Estimate(S.E.)	Estimate(S.E.)
**Structure**			
**STR1**	0,95(0,02)	0,95(0,02)	0,98(0,01)
**STR2**	0,72(0,08)	0,78(0,06)	0,79(0,06)
**STR3**	1,00(0,00)	1,00(0,00)	1,00(0,00)
**STR4**	0,86(0,06)	0,96(0,02)	0,98(0,01)
**Explication**			
**EXPL1**	1,00(0,00)	0,99(0,01)	1,00(0,01)
**EXPL2**	0,97(0,02)	0,96(0,02)	0,94(0,03)
**EXPL3**	0,97(0,01)	0,97(0,02)	0,98(0,01)
**EXPL4**	0,74(0,06)	0,87(0,04)	0,84(0,05)
**Stimulation**			
**STIM1**	1,00(0,00)	1,00(0,00)	0,99(0,01)
**STIM2**	0,93(0,02)	0,97(0,01)	0,95(0,02)
**STIM3**	0,97(0,01)	0,98(0,01)	0,97(0,01)
**STIM4**	0,99(0,01)	0,99(0,01)	1,00(0,01)
**Validation**			
**VAL1**	0,79(0,07)	0,81(0,05)	0,79(0,07)
**VAL2**	0,90(0,07)	0,94(0,02)	0,95(0,02)
**VAL3**	1,00(0,05)	0,98(0,02)	0,98(0,02)
**VAL4**	0,88(0,06)	0,97(0,02)	1,00(0,00)
**Instruction**			
**INSTR1**	0,96(0,06)	1,00(0,00)	0,88(0,08)
**INSTR2**	0,99(0,06)	0,95(0,02)	1,00(0,02)
**INSTR3**	0,54(0,14)	0,93(0,04)	0,94(0,04)
**INSTR4**	0,80(0,08)	0,96(0,03)	0,93(0,03)
**Comprehension**			
**COMP1**	0,93(0,04)	0,95(0,03)	0,92(0,03)
**COMP2**	0,95(0,03)	0,99(0,01)	0,97(0,02)
**COMP3**	0,80(0,07)	0,92(0,03)	0,90(0,03)
**COMP4**	0,93(0,04)	0,84(0,05)	0,94(0,03)
**Activation**			
**ACT1**	0,92(0,03)	0,95(0,03)	0,95(0,02)
**ACT2**	1,00(0,01)	0,99(0,02)	1,00(0,00)
**ACT3**	0,97(0,01)	0,97(0,02)	0,97(0,01)
**ACT4**	0,96(0,02)	0,97(0,03)	0,97(0,01)

Notes: At each measurement occasion the model contained a seven-factor model at the teacher level and an unrestricted model at the student level. Correction was made for Condition. Estimates and standard error are given on teacher level. T1 = measurement occasion one, T2 = measurement occasion two, T3 = measurement occasion three. S.E. = standard error.

Factor correlations on teacher level at each measurement occasion are presented in Table 7. Correlations among the seven factors varied from 0.22 to 0.93 (*M* = .52) at T1, from 0.36 to 0.94 (*M* = .66) at T2, and from .34 to .95 (*M* = .64) at T3. Overall, correlations tended to be high and showed a similar pattern over the measurement occasions.

**Table 7:** ISQ factor correlations—measurement occasion one, two and three.

**T1**	**Structure**	**Explication**	**Stimulation**	**Validation**	**Instruction**	**Comprehension**
**Explication**	0,84					
**Stimulation**	0,48	0,74				
**Validation**	0,66	0,70	0,67			
**Instruction**	0,81	0,69	0,44	0,71		
**Comprehension**	0,31	0,36	0,41	0,28	0,32	
**Activation**	0,22	0,32	0,48	0,34	0,27	0,94
**T2**	**Structure**	**Explication**	**Stimulation**	**Validation**	**Instruction**	**Comprehension**
**Structure**						
**Explication**	0,92					
**Stimulation**	0,76	0,89				
**Validation**	0,83	0,85	0,85			
**Instruction**	0,77	0,71	0,69	0,98		
**Comprehension**	0,36	0,36	0,47	0,45	0,44	
**Activation**	0,46	0,50	0,65	0,59	0,52	0,94
**T3**	**Structure**	**Explication**	**Stimulation**	**Validation**	**Instruction**	**Comprehension**
**Structure**						
**Explication**	0,88					
**Stimulation**	0,68	0,87				
**Validation**	0,81	0,84	0,86			
**Instruction**	0,83	0,73	0,67	0,83		
**Comprehension**	0,33	0,35	0,48	0,45	0,38	
**Activation**	0,38	0,47	0,60	0,58	0,46	0,95

Note: these factor correlations are on teacher level.

### Construct validity of the ISQ

We added the students’ self-assessed learning outcome variables Cognition, Affection and Regulation as dependent variables to the multilevel model to investigate the construct validity of the ISQ. The descriptive information on these three variables at T1 and T2 are presented in Table 8.

**Table 8:** Descriptive information on students’ perceptions of their learning outcomes—measurement occasion one and two.

Student Learning Outcomes	T1				T2			
	Teacher level		Student level		Teacher level		Student level	
	Mean	Variance	Variance	ICC	Mean	Variance	Variance	ICC
**Cognition**	4,99	0,18	1,33	0,12	5,07	0,26	1,32	0,12
**Affection**	4,35	0,27	1,85	0,13	4,37	0,35	1,78	0,16
**Regulation**	4,80	0,10	1,69	0,06	4,88	0,13	1,63	0,07

Notes: T1 = measurement occasion one. T2 = measurement occasion two. T3 = measurement occasion three. ICC = intra-class correlation

The fitted multilevel model included the seven-factor model at the teacher level, an unconstrained model at the student level, C1 and C2 as teacher level dummy coded covariates (to correct for the experimental design), and Cognition, Affection and Regulation as dependent variables at the teacher level. Table 9 shows the results of the regression analyses at T1 and T2 for each dependent variable on all the ISQ dimensions at the teacher level. We applied the Holm-Bonferroni step-down procedure to correct for multiple testing.

**Table 9:** Regression coefficients for ISQ dimensions on students’ perceptions of their learning outcomes—measurement occasion one and two.

		T1			T2		
Dependent variable	Independent variable	Estimate S.E.	Est./S.E.	p value	Estimate S.E.	Est./S.E.	p value
***Student level factors***	***Teacher level factors***						
**Cognitive Learning Outcome: “I learned a lot from this lecture”**	**Structure**	0,79(0,18)	4,29	0,00**	0,80(0,33)	2,46	0,01**
	**Explication**	-0,31(0,20)	-1,58	0,11	-0,56(0,38)	-1,47	0,14
	**Stimulation**	0,85(0,14)	6,06	0,00**	0,96(0,22)	4,40	0,00**
	**Validation**	-0,29(0,19)	-1,50	0,13	-0,03(0,15)	-0,18	0,86
	**Instruction**	0,10(0,19)	0,50	0,61	0,07(0,14)	0,52	0,60
	**Comprehension**	-0,60(0,34)	-1,76	0,08	0,19(0,29)	0,66	0,51
	**Activation**	0,49(0,36)	1,35	0,18	-0,40(0,34)	-1,19	0,24
**Affective Learning Outcome: “Because of this lecture, I want to learn more about the subject matter”**	**Structure**	0,25(0,18)	1,44	0,15	0,76(0,42)	1,80	0,07
	**Explication**	-0,40(0,19)	-2,10	0,04	-1,44(0,57)	-2,53	0,01**
	**Stimulation**	1,20(0,10)	11,47	0,00**	1,96(0,34)	5,79	0,00**
	**Validation**	-0,04(0,14)	-0,32	0,75	-0,09(0,25)	-0,38	0,71
	**Instruction**	-0,03(0,13)	-0,23	0,82	-0,13(0,22)	-0,57	0,57
	**Comprehension**	-0,11(0,30)	-0,37	0,72	0,38(0,38)	1,01	0,31
	**Activation**	0,01(0,30)	0,02	0,98	-0,50(0,43)	-1,16	0,25
**Regulative Learning Outcome: “Because of this lecture, I now know what I have yet to study”**	**Structure**	0,46(0,31)	1,50	0,13	0,50(0,37)	1,36	0,17
	**Explication**	-0,64(0,30)	-2,13	0,03	-0,53(0,46)	-1,15	0,25
	**Stimulation**	0,39(0,20)	1,95	0,05	-0,03(0,26)	-0,11	0,91
	**Validation**	-0,65(0,25)	-2,57	0,01**	-0,27(0,21)	-1,32	0,19
	**Instruction**	1,22(0,26)	4,67	0,00**	1,17(0,16)	7,52	0,00**
	**Comprehension**	-0,73(0,58)	-1,26	0,21	-0,04(0,53)	-0,08	0,94
	**Activation**	0,49(0,58)	0,84	0,40	-0,01(0,63)	-0,01	0,99

Notes: T1 = measurement occasion one, T2 = measurement occasion two. S.E. = standard error. Est. = Estimate. The tested multilevel regression model contained seven independent teacher level factors and three dependent student level variables. Corrections were made for Condition.

** regression coefficient is significant according to the Holm-Bonferroni step-down procedure on 21 tests per measurement occasion.

**Fig 2:**
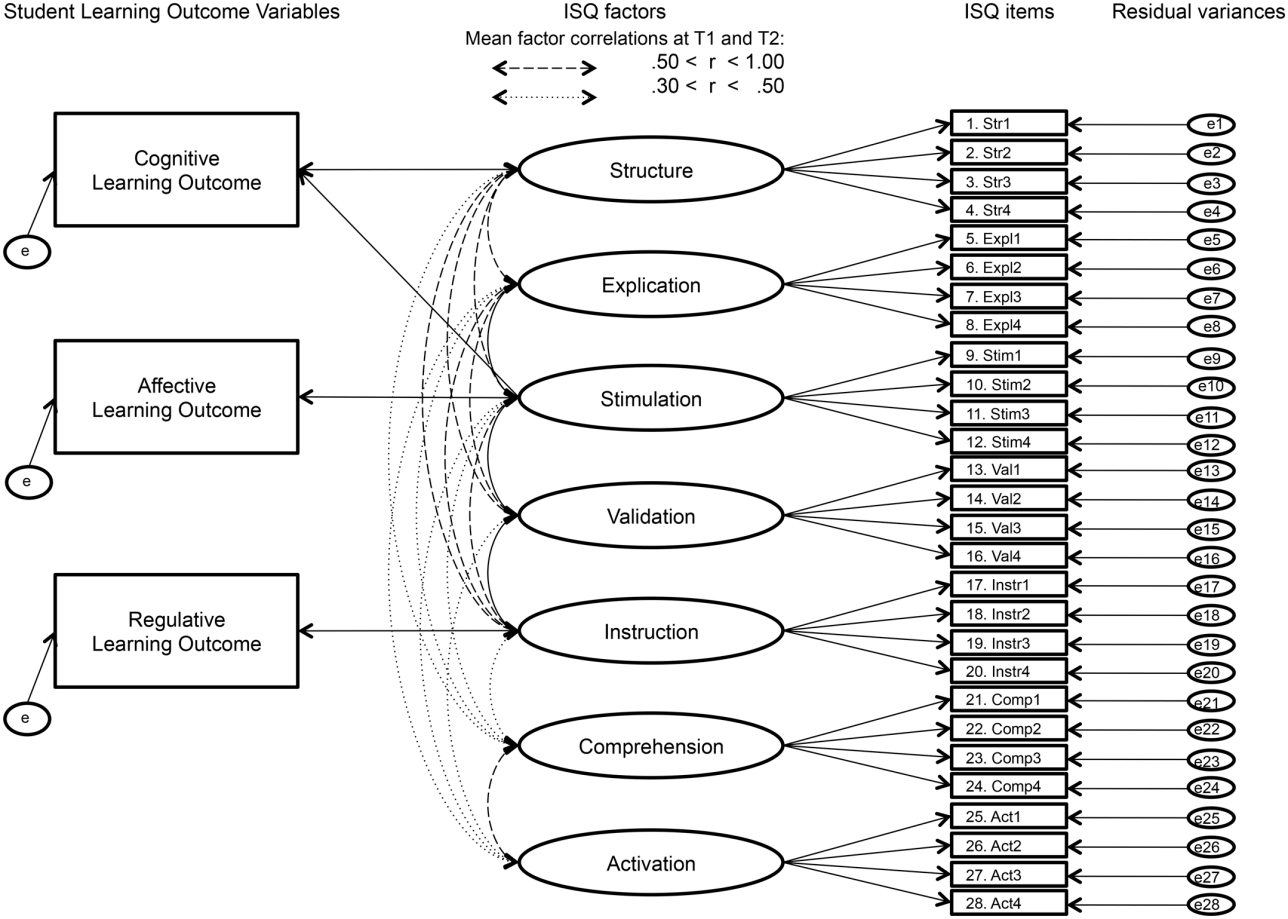
The relationship between ISQ dimensions and students’ perception of their learning outcomes–measurement occasion one and two.

At both measurement occasions, results showed significant effects of the factors Structure and Stimulation on the learning outcome variable Cognition, of the factor Stimulation on the learning outcome variable Affection, and of the factor Instruction on the learning outcome variable Regulation. A full representation of the final teacher level model and the relationship with students’ perceptions of their learning outcomes is given in Fig 2. In addition, there was a significant effect of Validation on Regulation at T1 and of Explication on Affection at T2. As these effects were not verified on both occasions, they were not represented in Fig 2.

## Discussion

Course evaluation instruments are often found to be a poor source of formative feedback for college professors concerning their teaching. The aim of the present study was to investigate the psychometric qualities of a new theory-based student evaluation of teaching instrument, the Instructional Skills Questionnaire (ISQ). This instrument was developed to assess detailed teaching behaviour, following each lecture. This way, the ISQ can be used to provide professors with immediate and specific feedback concerning their teaching. Our conceptualization of teaching in terms of the seven ISQ dimensions was based on the dimensions previously proposed in the literature [17, 23, 25], and on Feldman’s categories of teaching behaviour [16].

First, we provided the descriptive information on the ISQ data. The intra-class correlations (ICC) indicated that on average 15% of the item variance was between professors. As both the reliability of a single item is expected to be large and individual differences between students are often considerable, we consider the average ICC of .15 to be relatively high. This average ICC is comparable to the degree of clustering reported in other studies on student ratings (see for example [34]). The remaining proportion of item variance (student level) reflects measurement error and systematic differences between the ratings of the students in the same class. While the student level item variance was quite constant over the ISQ items, the teacher level variance varied considerably over the items. For example, professors did not display much variance from one another in their degree of structuring (ISQ dimension: Structure). However, they varied substantially with respect to degree to which they stimulated and activated students (ISQ dimension: Stimulation and Activation). This is reflected in the item intra class correlation increase. These differences in item variance on the teacher level are understandable, given a difference in level of difficulty between the ISQ dimensions (it is more difficult to engage and activate al large classroom than to structure the subject matter). This pattern of differences in teacher level variance on ISQ items was consistent over the three measurement occasions. Similarly student level item variances were consistent over the measurement occasions.

Next, we tested the reliability and factor structure of the seven ISQ dimensions of teaching behaviour at each measurement occasion. The teacher level reliabilities of the seven dimensions were found to be good (from .90 to .99) at each measurement occasion. One reason that these values were quite high is that the teacher scores were based on the average test scores of their students. The averages were necessarily subject to less measurement error variance than the student level data. In addition, the fit-indices of the multilevel confirmatory factor models indicated that the teacher level seven-factor model fitted the data adequately at each measurement occasion. Thus we conclude that the ISQ adequately measures seven dimensions of the professors’ lecturing behaviour. The instrument provided reliable and internally valid ratings on professors from a wide variety of departments at a Dutch university, on multiple occasions.

**Fig 3:**
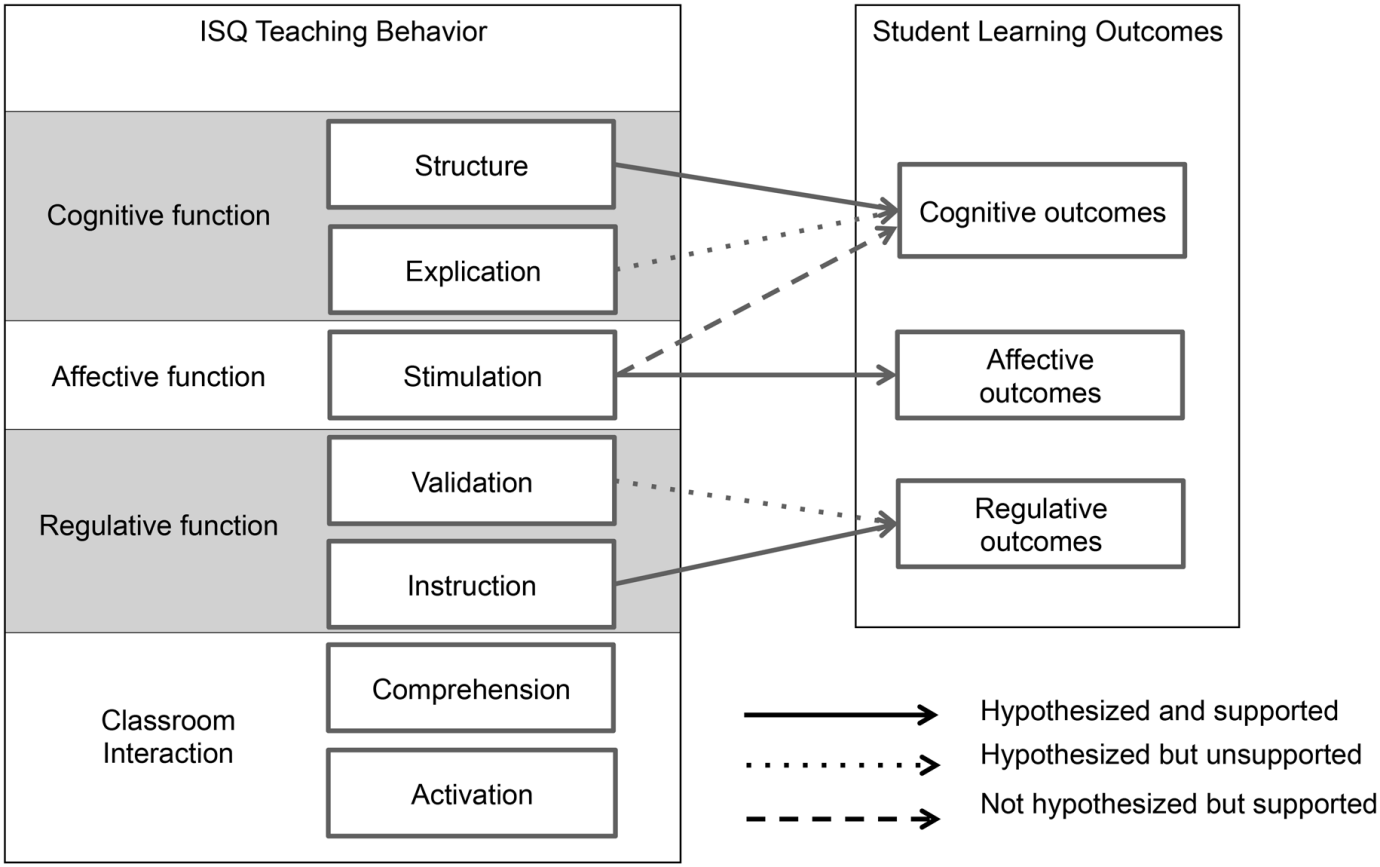
Conceptual framework on teaching behavior and student learning—supported and not supported hypotheses.

In the introduction, we proposed a theoretical framework (represented in Fig 1) on the relationship between teaching behaviour and the student learning process. We investigated this framework and the construct validity of the ISQ in exploratory analyses. With multilevel regression analyses we explored the relationship between ISQ dimensions and the dependent variables Cognition, Affection and Regulation (representing the students’ perceptions on their learning outcomes). Overall, the findings (represented in Fig 2) provide partial support for the proposed theoretical framework. The supported and not supported hypotheses are visualized in Fig 3.

While we had not anticipated the direct effect of the factor Stimulation on the cognitive learning outcome variable, it is consistent with Feldman [16]. Specifically, Feldman related teaching dimensions to domains of student achievement and overall evaluations. He found the dimensions teacher comprehensibility and clarity (identical to the dimensions Structure and Explication) and teacher stimulation of interest in the subject matter (identical to the dimension Stimulation) to be highly related to student achievement and overall teacher ratings. The direct relationships found between ISQ teaching dimensions and student learning outcome variables further support the construct validity of the instrument.

A final point to address is the correlations between the ISQ factors. We note that at the teacher level several ISQ factors correlated from 0.22 to 0.93 with a mean of .52, with a few correlation exceeding .8. Relatively high factor correlations are characteristic of many SET instruments. For example, confirmatory factor analysis of the SEEQ instrument resulted in factor correlations ranging from .02 to .87 with a median of .72 [41]. From the perspective of content validity, we maintain that it is better to retain factors even if they are quite highly correlated. For example, Comprehension and Activation correlate highly, but the teaching goals differ (Comprehension: to check whether students understand the subject matter, versus Activation: to activate students in the lecture). Retaining these as distinct factors also guarantees the specificity of the feedback to professors, which is a major objective of this instrument. In addition, as explained in the methods section, the ISQ was used in an experimental design in which professors did or did not receive intermediate feedback and consultations in between the measurement occasions to improve their instructional skills. While the full results are beyond the present scope, we note that the findings showed different significant effects of consultation on ISQ dimensions, notwithstanding their correlations (e.g. Comprehension and Activation). Again, this indicated the discriminant validity of the dimensions. Finally, while a small number of correlation are large, we are reticent to collapse factors as the relatively high correlations may be a characteristic of the population studies, and need not generalize to other populations of lecturers, teachers and professors.

In sum, this study offers a reliable and valid instrument to evaluate single lectures. The instrument was based on extensive research on student evaluation of teaching to ensure the instrument’s content validity. The internal structure, reliability, and construct validity of the ISQ teaching dimensions were demonstrated to be adequate.

In terms of future directions, we suggest using student ratings of teaching (like the ISQ ratings) more often as (intermediate) formative feedback instruments instead of summative instruments. Further investigations on various ways to use student ratings in a formative manner, as for example mid-term feedback [5] or combined with peer-coaching or expert consultation [6, 7], are more likely to have an impact on teaching quality. Such investigations and practices in the field would serve the initial main purposes of collection student ratings: to help professors improve their own teaching practices as well as student learning outcomes.

Using the ISQ, one can provide professors with immediate, specific and reliable feedback on their teaching and on differences between student learning outcomes during a course. In addition, researchers can use the ISQ to measure differences between professors in teaching behaviour. Finally, the ISQ provides new insights into the classroom dynamics that characterize university lectures. Even though the lecture is not the most popular teaching format, it remains in extensive use at universities worldwide. This study shows that professors have a direct influence on how useful a lecture actually is in terms of the student learning process.

## Supporting Information

S1 Table Dutch version of the Instructional Skills Questionnaire (ISQ). (XLSX)

S2 Table Class size per professor per measurement occasion. (XLSX)

S3 Table ISQ reliability on student level—measurement occasion one, two and three. (XLSX)
